# Surgical outcome and predictors of neonates with esophageal atresia admitted at Tikur Anbesa Specialized Hospital

**DOI:** 10.1371/journal.pone.0285669

**Published:** 2023-05-16

**Authors:** Natnael Moges, Kassaye Ahmed, Dires Birhanu, Fekadesellasie Belege, Asrat Dimtse, Gashaw Kerebeh, Belayneh Dessie Kassa, Kumlachew Geta, Keder Essa Oumer, Edgeit Abebe Zewde, Anteneh Mengist Dessie, Denekew Tenaw Anley, Solomon Demis, Fisha Alebel GebreEyesus, Berihun Bantie

**Affiliations:** 1 Department of Paediatrics and Child Health Nursing, Debretabor University, College of Health Science, Debre Tabor, Ethiopia; 2 Department of Neonatal Nursing, University of Gondar, College of Health Science, Gondar, Ethiopia; 3 Department of Paediatrics and Child Health Nursing, Dilla University, College of Health Science, Dilla, Ethiopia; 4 Department of Paediatrics and Child Health Nursing, Wollo University, College of Health Science, School of Nursing and Midwifery, Dessie, Ethiopia; 5 Department of Neonatology, Addis Ababa University, College of Health Science, School of Medicine, Addis Ababa, Ethiopia; 6 Debretabor University, College of Health Science, School of medicine, DebreTabor, Ethiopia; 7 Department of Aesthesia, Debretabor University, College of Health Science, Debretabor, Ethiopia; 8 Department of Biomedical Science, Debretabor University, College of Health Science, Debretabor, Ethiopia; 9 Department of Public Health, Debretabor University, College of Health Science, Debretabor, Ethiopia; 10 Department of Department of Paediatrics and Child Health Nursing, Wolkite University, College of Medicine and Health Sciences, Wolkite, Ethiopia; 11 Department of Adult Health Nursing, Debretabor University, College of Health Science, Debretabor, Ethiopia; Cairo University Kasr Alainy Faculty of Medicine, EGYPT

## Abstract

**Background:**

Esophageal atresia (EA) with or without tracheoesophageal fistula (TEF) is the most common congenital anomaly of the esophagus. This anomaly continues to cause considerable morbidity and mortality in Sub-Saharan Africa, presenting various concerns about how to treat esophageal atresia. Esophageal atresia-related neonatal mortality can be reduced by evaluating the surgical outcome and identifying associated factors.

**Objective:**

This study aimed to assess the surgical outcome and identify predictors of neonates with esophageal atresia admitted at Tikur Anbesa specialized hospital.

**Methods:**

Retrospective crossectional study design was employed on 212 neonates with esophageal atresia who were undergone surgical intervention in Tikur Anbesa specialized hospital. Data were entered into epi data 4.6 and exported to Stata version 16 software for further analysis. A logistic regression model with Adjusted odds ratio (AOR), confidence interval (CI) and p-value <0.05 were used to identify predictors of poor surgical outcome of neonates with esophageal atresia.

**Result:**

In this study, 25% of newborns who underwent surgical intervention at TikurAbnbesa specialized hospital had successful surgical outcomes, compared to 75% of neonates with esophageal atresia who had poor surgical outcomes. Significant predictors of the poor surgical outcome of neonates with esophageal atresia were severe thrombocytopenia (AOR = 2.81(1.07–7.34)), timing of surgery (AOR = 3.7(1.34–10.1), aspiration pneumonia (AOR = 2.93(1.17–7.38)) and related abnormalities (AOR = 2.26(1.06–4.82)).

**Conclusion:**

The results of this study showed that, when compared to other studies, a substantial percentage of newborn children with esophageal atresia had poor surgical outcomes. Early surgical management, aspiration pneumonia and thrombocytopenia prevention and therapy play a big part in improving the surgical prognosis for newborns with esophageal atresia.

## Introduction

Thomas Gibson first described esophageal atresia (EA) associated with tracheoesophageal fistula (TEF) in 1697. However, it was not until 1941 that Cameron Haight performed the first successful surgical repair of this anomaly following innumerable attempts by other surgeons [1]. Esophageal atresia (EA) with or without tracheoesophageal fistula (TEF) is the most common congenital anomaly of the esophagus [2].

The overall incidence of EA/TEF ranges from one in every 2500 to 4500 live births [3]. The vast majority of cases are sporadic, although the incidence is higher in twins. It has been reported that the relative risk for EA/TEF in twins was 2.56 higher than in singletons [3] Of the total neonates with EA, 93% to 94% present with a combination of both TEF and EA, while approximately 6% to 7% of newborns manifest with only EA [4]. Determining the appropriate surgical and medicinal strategy in this population requires an understanding of these anatomical abnormalities [2]. Surgery is the only treatment option for newborns with esophageal atresia [5]. However, pediatric surgical care for this complex deformity is still challenging [1,2].

In industrialized nations, esophageal atresia is regarded as a deadly defect and neonatal emergency with a markedly improved prognosis. Because of improvements in medical care, especially neonatal and surgical procedures, high-income countries’ survival rates now surpass 95%, and a growing proportion of EA neonates grow up [6,7]. Additionally, EA newborns without accompanying malformations were reported to have 100% one-week survival rates [8,9]. For EA neonates delivered with concomitant cardiac abnormalities, very low birth weight (1,500 g), and long-gap EA, lower rates of up to 87% have been documented [10,11].

Mortality rates from EA range from 30 to 80% in low- and middle-income countries (LMIC) [12]. This anomaly continues to cause considerable morbidity and mortality in Sub-Saharan Africa, presenting various concerns about how to treat esophageal atresia [13,14].

Esophageal atresia has a positive prognosis in industrialized nations, whereas the opposite is true in developing nations. Ethiopia is seeing an increase in the number of newborns with EA cases, and the majority of these infants have passed away for various reasons either before receiving surgical treatment or just after it [7,15].

Esophageal atresia accounted for 3% of intrauterine fatalities and 27% of induced abortion among perinatally identified pregnancies [16,17]. However, live birth was a factor in the majority of EA cases. Anomalies or birth abnormalities are present in about 55% of EA live births [4].

Most significantly, there has never been a study done before about surgical outcomes and predictors among Ethiopian EA newborns. To help the nation, prevent neonatal mortality attributable to esophageal atresia and help meet the neonatal target of the Sustainable Development Goals (SDG) by 2030, this study sought to identify the outcome status and predictors among neonates with the condition in Tikur Anbesa Specialized Hospital (TASH).

## Methods

### Study setting, design, period, and population

Between March 1, 2011, and February 30, 2021, EA newborns admitted to the NICU at Tikur Anbesa Specialized Hospital (TASH) were the subject of an institutionally based retrospective cross-sectional investigation. The headquarters of the African Union and the UN World Economic Commission for Africa are located in Addis Abeba, the capital city of Ethiopia. TASH was founded in early 1964 and is the biggest and most well-known public hospital. TASH is the single largest specialized hospital in Ethiopia, and it is the only hospital that serves as a management center for neonates having esophageal atresia in the country. All medical charts of surgically treated neonates with esophageal atresia at TikurAnbesa Specialized Hospital during the study period were the source population. Data were collected from February 10 to March 10 /2021.

### Eligibility criteria

All medical charts of surgically treated neonates with esophageal atresia at Tikur Anbesa Specialized Hospital from March 1/2011- February 30/2021 were included. Medical charts with incomplete information (discharge date and outcome status) were excluded from the study.

### Sample size determination and sampling procedure

The sample size was determined by using single population proportion formula, 95% confidence interval, 5% margin of error, and the proportion of surgical outcome of 50% (because there is no prior study). The required sample size (N) is 385. Since the source population is less than 10,000, we used the sample size correction formula and the sample size is reduced to 212. Neonates with surgical intervention for EA from the NICU registration book were selected randomly and enrolled in the study.

### Operational definition

**Poor surgical outcome:** neonates with EA who died after undergoing surgical intervention. **Good surgical outcome**–neonates with EA who had improvement and discharged from the hospital after surgical intervention. **Malnutrition:** Neonates with EA whose serum albumin level is low to age or kept NPO for a prolonged time or edematous and documented on the chart as malnutrition. **Dehydration:** Neonates with EA who had signs and symptoms of dehydration and documented on the medical chart as dehydration. **Sepsis:** Neonates with EA who had signs and symptoms of infection and documented on the chart as sepsis [15].

### Data collection procedure and tool

The data extraction format was created in English to extract all of the pertinent data from the records of EA neonates after thorough verification of all information that was available in those records. From the registration records, all of the charts of surgically treated infants with EA admitted to TASH between March 2011 and February 2021 were retrieved, and the charts of all study participants were chosen based on the qualifying requirements. The data was collected by certified NICU nurses.

### Data quality assurance and analysis

Supervisors and data collectors received one day of training on how and what information they should gather from the intended data sources to maintain data quality. Before data collection, 11 (5% of the study subjects) were used to examine (pretest) data extraction forms for consistency and completeness. We used Cronbach’s Alpha test and pilot testing to check the questions’ internal consistency. All of the questions were subjected to a Cronbach’s Alpha test, and the results showed strong internal consistency (71%).

Data were entered by Epi-data (version 4.6.0.2) and exported to Stata (version 16) for further analysis. All variables with a p-value ≤ 0.25 were taken into the multivariable logistic regression model to control for all possible confounders. A logistic regression model with an adjusted odds ratio (AOR), 95% confidence interval (CI) and p-value of ≤ 0.05 was used to identify predictors of the outcome variable.

The overall goodness of the model with the fitted data was evaluated using the "Hosmer and Lomeshow" goodness of fit test. Additionally, the discriminative power of the model was examined using the receiver operating characteristic curve (ROC curve), which is created by plotting the true positive rate against the false positive rate of the model at different thresholds. An area under the curve (AUC) value of greater than 70% was then used to classify the model as being able to accurately classify people with and without the outcomes.

#### Assumption of binary logistic regression

**Fig 1:**
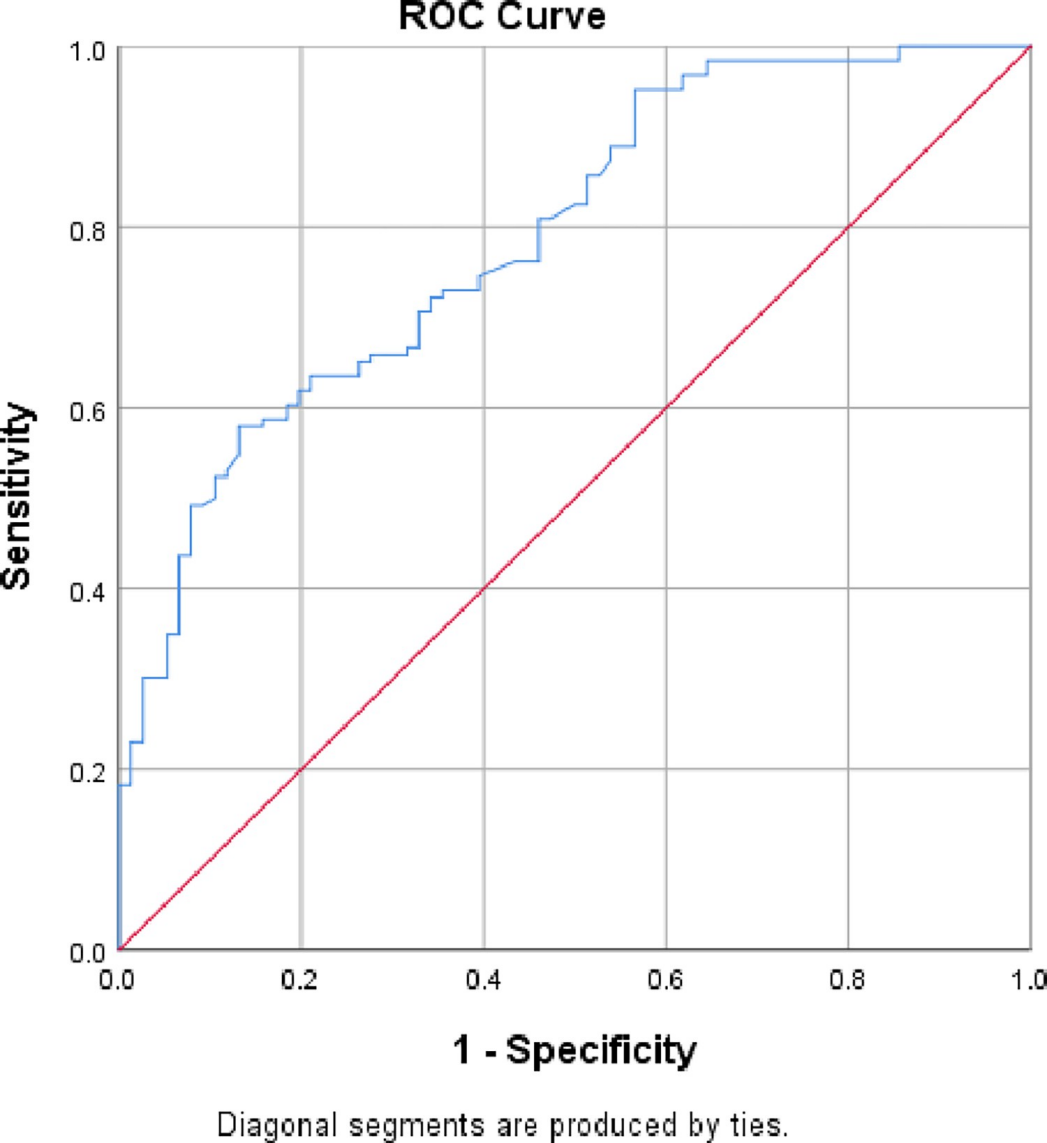
ROC curve graph showing model discrimination power towards surgical outcome and associated factors of neonates with esophageal atresia admitted at Tikur Anbesa Specialized Hospital, Addis Ababa, Ethiopia,2021.

According to the Hosmer and Lomeshow goodness of test for multivariable logistic regression, the model made up of such factors is capable of accurately predicting the surgical result outcome variable (P-value = 0.471). In fact, the receiver operating curve was used to test the model’s discrimination abilities, and the area under the curve (AUC value = 0.788) showed that the model performs exceptionally well at differentiating between people who have good surgical outcomes and those who have poor surgical outcomes (true positives) (**Fig 1)**.

### Ethics statement

This study was approved by the research ethics committee from Addis Ababa university college of health science, school of nursing, and informed consent was exempted because we only accessed identified previously collected data. The data could be accessed with the permission of Tikur Anbesa Specialized Hospital.

## Result

### Sociodemographic and neonatal-related factors of the study participants

In this study, 212 cases have sufficient data for inclusion. Among 212 study participants,96.2% were referral cases. Regarding the time of diagnosis,59.1% of surgically managed neonates were diagnosed after two days of admission. Among the participants of the study,88.2% of neonates with oesophageal atresia have proximal esophageal atresia with distal TEF (type C) oesophageal atresia (Table 1).

**Table 1:** Socio-demographic and neonatal conditions of neonates with esophageal atresia admitted to TikurAnbesa Specialized Hospital, Addis Ababa, Ethiopia; from March 2011 to February 2021 (n = 212).

Socio-demographic and neonatal characteristics	Category	Frequency (%)
Admission source	Inborn	8(3.8)
	Out born	219(96.2)
Postnatal age at admission	<1day	52(24.5)
	1-2day	24(11.3)
	>2day	136(64.2)
Gestational age (GA)	28–36 week(wk.)	66(31.1)
	37-42wk	146(68.9)
Sex	Male	104(49.1)
	Female	108(50.9)
Weight	2500–4000 gram(gm)	140(66)
	<2500g	72(33)
Type of variants (n = 203)	Type A	10(4.7)
	Type B	3(1.4)
	Type C	187(88.2)
	Type D	3(1.4)
Time of diagnosis	< 48 hours(hrs)	92(40.9)
	>48 hr	133(59.1)
Associated congenital anomalies	Yes	85(40.1)
	No	133(59.9)

#### Clinical comorbid related factors of poor surgical outcome of neonates with EA

Sepsis 112(52.8%), aspiration pneumonia 174(82.1%), malnutrition 140(69.3%), thrombocytopenia 166(79.1%) and dehydration 34(16%) were among the clinical comorbid conditions in surgically treated neonates with esophageal atresia.

#### Management-related factors of poor surgical outcome of neonates with EA

Primary repair (fistula ligation and end-to-end esophageal anastomosis) was done for 184(86.8%) of the EA neonates while gastrostomy was done for 28(13%) of the EA neonates. Among those EA neonates who were treated by surgical intervention, 36(17.1%) had surgery within three days of admission whereas 175(82.9%) had surgery after three days of admission. About 40(18.9%) of the EA neonates had a surgical-related complication and,10(8.4%) of whom had an anastomotic leak while only 3(2.5%) had surgical site infection.

#### Factors associated with the poor surgical outcome of neonates with Esophageal atresia

All variables were undergone bivariable logistic regression. Gestational age, birth weight, time of diagnosis, associated anomalies, malnutrition, aspiration pneumonia, thrombocytopenia, surgical related complication, dehydration and time of surgery were run to multivariable logistic regression for further analysis. In multivariable logistic regression, associated anomalies, severe thrombocytopenia, time of surgery and aspiration pneumonia were significantly associated with the poor surgical outcome of neonates with EA (Table 2).

**Table 2:** Bivariable and multivariable logistic regression model for factors of poor surgical outcome of neonates with esophageal atresia admitted in NICU at TikurAnbesa Specialized Hospital from March 2011 to February 2021(n = 225).

Factor	Outcome		COR (95%CI)	AOR (95% CI)	P-value
	Good (%)	Poor (%)			
**Gestational age**					
Term	68(32.1)	78(36.8)		1	
Preterm	12(5.7)	54(25.4)	3.92(1.94–7.94)	2.18(0.86–5.51)	0.100
**Birth weight**					
NBW	64(30.3)	76(35.8)	1	1	
LBW	16(7.5)	56(26.4)	2.95(1.54–5.63)	2.61(1.07–6.4)	0.036
**Diagnosis time**					
<48 hr	38(17.9)	70(33.1)	1	1	
>48 hr	42(19.8)	62(29.2)	0.8(0.46–1.4)	0.36(0.17–0.76)	0.008
**Associated anomalies**					
No	58(27.4)	69 (32.5)	1	1	
Yes	22(10.4)	63(29.7)	2.40(1.32–4.38)	**2.26(1.06–4.82)**	0.035*
**Time of fistula repair**					
<72hr	25(11.8)	11(5.7)	1	1	
>72hr	55(25.9)	120(56.6)	4.96(2.28–10.79)	**3.7(1.34–10.2)**	0.012*
**Sepsis**					
No	44(20.8)	56(26.4)	1	1	
Yes	36(17)	76(35.8)	1.66(0.95–2.90)	1.91(0.93–3.94)	0.078
**Aspiration pneumonia**					
No	24(11.3)	14(6.6)	1	1	
Yes	56(26.4)	118(55.7)	3.61(1.74–7.50)	**2.93(1.17–7.38)**	0.022*
**Malnutrition**					
No	30(14.9)	32(15.8)	1	1	
Yes	46(22.8)	94(46.5)	1.92(1.04 3.53)	2.81(1.3–6.1)	0.008
**Thrombocytopenia**					
150k-350k	16(7.5)	20(9.4)	1	1	
100-149k	8(3.8)	6(2.8)	0.6(0.17–2.09)	2.184.874	0.298
50-99k	10(4.8)	22(10.4)	1.76(0.65–4.76)	245.468	0.177
<50k	46(21.7)	84(39.6)	1.46(0.69–3.09)	**2.81(1.07–7.34)**	0.037*
**Dehydration**					
No	72(33.9)	106(50)	1	1	
Yes	8(3.8)	26(12.3)	2.21(0.95–5.15)	1.82(0.62–5.34)	0.272
**Surgical related complication**					
No	70(33)	102(48.2)	1	1	
Yes	10(4.7)	30(14.1)	2.06(0 .95–4.48)	2.37(0.88–6.34)	0.086

1-reference.

*Significant association with outcome of neonates with esophageal atresia.

Esophageal atretic neonates with associated anomalies increase the risk of the poor surgical outcome by 2.26 times (AOR = 2.26(1.06–4.82)) compared to their counterparts.

Surgical treatment after three days of admission of neonates with esophageal atresia increases the risk of poor surgical outcome by 3.7-fold (AOR = 3.7(1.34–10.2)) compared to newborn infants who underwent surgery before three days of admission.

The odds of poor surgical outcome among neonates with EA who have severe thrombocytopenia is 2.81 times (AOR = 2.81(1.07–7.34)) higher compared to those without severe thrombocytopenia.

Newborn babies diagnosed with Esophageal atresia and aspiration pneumonia increase the chance of poor surgical outcome by 2.93times (AOR = 2.93(1.17–7.38)) than neonates without aspiration pneumonia.

## Discussion

This study aimed to determine the surgical outcome and associated factors of neonates with esophageal atresia. In this study, 75% with 95% CI of (68%- 80%) of neonates have died after undergoing surgical intervention. This finding is comparable with the study conducted in Algeria [6] and Senegal [14] but higher than studies conducted in the France [18]and Tunisia [19]. The difference could be explained by the advanced surgical and medical care provided to newborns with esophageal atresia in western nations. Additionally, one factor contributing to the greater fatality rate in the TASH context may be the lack of complete parenteral nutrition (TPN) and mechanical ventilation in the NICU of TASH [15].

In this finding, associated anomalies, time of surgery, severe thrombocytopenia and aspiration pneumonia were among the factors associated with the poor surgical outcome of neonates with esophageal atresia.

The odds of poor outcomes among neonates with EA having associated anomalies is 2.26 times higher compared to neonates without associated anomalies. This is supported by clinical evidence that esophageal atretic neonates with associated congenital anomalies could develop several complications which lead the infants to deterioration and poor prognosis. This finding is also supported by the study conducted in Columbus [11] and Serbia [20]. The presence of associated congenital anomalies can have a significant impact on the surgical outcome of neonates with esophageal atresia. These anomalies can include, but are not limited to, tracheoesophageal fistula, cardiac defects, and abnormalities of the central nervous system. These additional anomalies can increase the complexity of the surgery and recovery period, and can also increase the risk of complications and long-term morbidity [21]. For example, infants with esophageal atresia and tracheoesophageal fistula typically require multiple stages of surgery, and infants with associated cardiac defects may require surgical intervention for those defects before or in conjunction with the surgery for esophageal atresia.

Time of fistula repair was found to have an association with poor outcomes of surgically treated neonates with esophageal atresia. Neonates with esophageal atresia who were treated with surgical intervention after three days of admission increased the chance of having a poor prognosis of neonates with esophageal atresia by 3.7-fold. This is supported by clinical evidence that newborn babies with esophageal atresia should be treated by surgical intervention within two days of hospital admission. Despite this, delayed surgical intervention will lead the babies to acquire different comorbid illnesses like aspiration pneumonia, sepsis, malnutrition and hematologic problems which can cause poor prognosis for surgically managed babies. This is supported by the research conducted in Serbia [20] and the USA [22].

In neonates with oesophageal atresia who were surgically managed, aspiration pneumonia was found to have an association with poor surgical outcomes. Aspiration pneumonia has increased the risk to have bad outcomes by 2.95times when compared to counterparts. Clinical evidence suggests that aspiration pneumonia is an especially dangerous complication of esophageal atresia in neonates due to the increased risk of impaired oxygenation and sepsis. Aspiration during anesthesia may cause the worst prognosis in patients who had undergone corrective surgery for esophageal atresia, leading to recurrent respiratory failure, aspiration pneumonia and worse outcomes. This finding is supported by in the studies conducted in Serbia [20] and Saudi Arabia [23].

Thrombocytopenia has a significant association with bad surgical outcome of neonates with esophageal atresia. It increases the probability of having a bad surgical outcome by 2.81-fold. Clinical evidence suggests that thrombocytopenia, or a low platelet count, can contribute to poor surgical outcomes in neonates with esophageal atresia. Platelets are important for blood clotting, and a low platelet count can lead to increased bleeding during and after surgery. This can prolong surgical time, increase the risk of complications, and lead to a higher rate of re-operation. Since, in babies with severe thrombocytopenia, performing timely surgical intervention would be challenging so, before surgical intervention is done, thrombocytopenia has to be managed. This finding is lower than the study conducted in Indonesia [24]. The discrepancy could be methodological difference.

In conclusion, the proportion of death among surgically treated neonates with esophageal atresia is found to be higher compared to studies. Associated congenital anomalies, aspiration pneumonia, time of surgery and thrombocytopenia were factors associated with poor outcomes of surgically treated neonates with esophageal atresia. early surgical intervention, prevention and management of aspiration pneumonia, and thrombocytopenia will be helpful to decrease the mortality of neonates related to esophageal atresia.

The strength of this study is providing a piece of baseline information for future studies conducted in this area. In addition, this study will have a role to decrease neonatal mortality due to esophageal atresia. On the contrary, this study has also limitations; the study design used in this study is crossectional which is difficult to determine the cause-and-effect relationship between the outcome and independent variables. Furthermore, the data collection was based on the chart review which is challenging to gather all the required information to do the study.

## Supporting information

S1 Data set (DTA)
